# Healthcare decision-making capacity in old age: A qualitative study

**DOI:** 10.3389/fpsyg.2022.1024967

**Published:** 2022-10-24

**Authors:** Ana Saraiva Amaral, Mário Rodrigues Simões, Sandra Freitas, Manuela Vilar, Liliana Baptista Sousa, Rosa Marina Afonso

**Affiliations:** ^1^Faculty of Psychology and Educational Sciences, University of Coimbra, Coimbra, Portugal; ^2^Center for Research in Neuropsychology and Cognitive and Behavioral Intervention (CINEICC), Faculty of Psychology and Educational Sciences (FPCE-UC), University of Coimbra, Coimbra, Portugal; ^3^Psychological Assessment and Psychometrics Laboratory (PsyAssessmentLab), Faculty of Psychology and Educational Sciences (FPCE-UC), University of Coimbra, Coimbra, Portugal; ^4^Faculty of Health Sciences, Health Sciences Research Center, University of Beira Interior, Covilhã, Portugal; ^5^The Health Sciences Research Unit, Coimbra Nursing School, Coimbra, Portugal; ^6^Department of Psychology and Education, Faculty of Social and Human Sciences, University of Beira Interior, Covilhã, Portugal; ^7^CINTESIS@RISE, School of Medicine and Biomedical Sciences (ICBAS), University of Porto, Porto, Portugal

**Keywords:** decision-making, healthcare, capacity assessment, older adults, focus groups

## Abstract

**Objectives:**

Research about decision-making capacity has been growing in the last decades. That relates to more concerns regarding patients’ autonomy, and an increase in diseases that can negatively impact capacity. This research aims to: explore perceptions, legal aspects, and assessment procedures related to healthcare decision-making capacity in older adults with cognitive impairment; and study the first version of a new assessment instrument of this capacity.

**Method:**

Nine focus groups were conducted, including healthcare, law and justice, nursing home professionals, institutionalized older adults, and dwelling older adults. Focus group discussions followed semi-structured interview scripts, specifically developed for each group. After group discussions, the assessment instrument was presented, and participants were asked to evaluate each item relevance and comprehensibility. Qualitative coding of the transcriptions was performed with resource to MAXQDA, using direct content analysis.

**Results:**

Six primary themes emerged from the qualitative analysis: Decision-making capacity features; Abilities implied in decision-making; Factors influencing decision-making; Obstacles to decision-making; Legal aspects; and Assessment procedures.

**Discussion:**

Results corroborate previous theoretical formulations of capacity. Generally, research results have implications for clinical and assessment practices, as well as preventive strategies that can improve older adult’s decision-making capacity. Assessment procedures of capacity should include a thorough protocol for the assessment of cognition, functionality, depressive symptoms, and decision-making abilities. In this respect, the need for an assessment tool that can provide valid information during evaluation processes is highlighted. Concerning the strategies to promote decision-making capacity, these rely on improving older adult’s health literacy and healthcare providers communication skills, as well as conduct actions to reduce stigma toward people with dementia.

## Introduction

Dementia represents a general term used to describe a significant decline in cognition, with a profoundly negative impact on the person’s functionality (Plassman and Potter, 2018). Dementia prevalence has been consistently increasing in the last decades. Currently, it is estimated that 55 million people have dementia worldwide, with approximately 10 million new cases per year. Considering the increasing prevalence of the older population, it is expected that in 2050 139 million people will suffer from dementia. Presently, dementia is among the major causes of dependency in older adults. The most frequent form of dementia is Alzheimer’s disease, which accounts for 60%–70% of all cases (World Health Organization, 2021). In the initial phases, individuals show signs of mild cognitive impairment, during which they can compensate for cognitive decline and function independently. In later stages, people present progressive cognitive impairment and increased dependency on daily functioning (Plassman and Potter, 2018).

In parallel, during the last decades, we have assisted to an increased concern regarding mental and civil capacity issues. Civil capacities refer to multiple domains required to maintain an autonomous and independent life. Initially, research in this field was encouraged by the deinstitutionalization movement (Moye et al., 2013). However, populations aging raised new questions in the research capacity field (Moye and Marson, 2007). Specifically, the increase of diseases associated with older age such as dementia upbrought the need to develop tools to evaluate capacity in this population (Triebel et al., 2018). During dementia progression, decision-making capacity is impaired and loss, which has ethical and legal repercussions for people with dementia, health providers, caregivers, and society in general (Triebel et al., 2018). In this study field, one of the biggest concerns is the assessment of capacity to give informed consent in healthcare. Older adults often face multi comorbidities, and the diagnosis of mild cognitive impairment or dementia does not necessarily imply a lack of capacity (Leonard, 2020). Therefore, the assessment of capacity to make decisions about healthcare is needed to guarantee the right of autonomy for people who retain capacity (Appelbaum, 2007), as well as support and protect those with impaired capacity (Palmer et al., 2012; Morris, 2020).

Healthcare decision-making capacity assessment should include medical history, clinical interview, neuropsychological and capacity assessment, with a specific tool (Moro et al., 2020). The inclusion of a specific tool is particularly important since it adds rigor and objective data regarding capacity (Triebel et al., 2018). There have been some tools specifically developed to evaluate this capacity in people with dementia, such as the Capacity to Consent to Treatment Instrument (CCTI; Marson et al., 1995) and the Assessment of Capacity to Consent to Treatment (ACC-T; Jennifer Moye et al., 2007). Although not specifically developed for people with dementia, the MacArthur Competence Assessment Tool for Treatment (MacCAT-T; Grisso and Appelbaum, 1998b) has also been used in studies with this population (Triebel et al., 2018). These instruments seem to share the same theoretical framework, since they evaluate four common abilities, with CCTI assessing a fifth ability (Amaral et al., 2021). Despite that, they show inconsistencies in how these abilities are assessed, which negatively impacts instruments’ reliability. Furthermore, previous studies that compared results from two of these instruments showed fragilities regarding concurrent validity (Moye et al., 2004). The four abilities common to these assessment instruments have been presented by Grisso and Appelbaum (1998a), who based their theoretical framework on the legal standards of competence to consent. Grisso and Appelbaum (1998a) propose that capacity to make health decisions rely on the person’s ability to understand, appreciate, reason, and communicate a choice.

In what concerns the Portuguese context, according to the Portuguese Penal Code, medical-surgical treatments must be preceded by patients’ informed consent (Decree-law 48/95, 1995). The patient’s consent is valid if they had been previously informed about the diagnosis, nature, extent, and possible consequences of the treatment. Furthermore, consent is valid when the patient has the necessary discernment to appreciate the treatment’s scope and meaning (Decree-law 48/95, 1995). However, what specific abilities the patient must have to discern about proposed treatments are not detailed, nor how they should be assessed. Circumstances of impaired capacity fall under the Legal Regime of the Accompanied Adult (Decree-law 49/2018, 2018), which stipulates that the specific acts in which the adult needs to be accompanied or substituted in decision-making are judicially defined (Beleza, 2019).

There are no assessment instruments of consent capacity for the Portuguese population. To compensate this need, and considering the fragilities of previous instruments identified above, the research team decided to develop a new assessment instrument. Therefore, this study presents a qualitative study on healthcare decision-making capacity, as part of a project that aims to develop a clinical assessment tool of this capacity, valid for older adults with neurodegenerative disorders like mild cognitive impairment and Alzheimer’s Disease, named Capacity Assessment Instrument—Health (CAI-Health). This instrument will include three clinical vignettes, a capacity assessment interview (to be conducted after each vignette presentation), and a questionnaire of healthcare values and preferences.

The development of the capacity interview was based on a theoretical revision, as well as previous assessment instruments available by the authors. The capacity assessment interview follows a structured script with open-ended questions. It has as theoretical framework Grisso and Appelbaum’s (1998a) four abilities model, allowing the assessment of understanding, appreciation, reasoning, and communicating a choice as operationalized by the authors. According to the same, understanding relates to the ability to understand information regarding health issues, treatment options, and its risks and benefits. Appreciation refers to the person’s ability to apply received information to their situation. On what concerns reasoning, authors describe it as the ability to weigh risks and benefits from each treatment option, to reach a decision. At last, expressing a choice regards the ability to communicate a decision clearly and consistently (Grisso and Appelbaum, 1998a).

The healthcare values and preferences questionnaire followed the same steps, regarding the revision of theoretical frameworks and previous assessment instruments. This questionnaire allows the assessment of the following variables: the desire for family and health professional involvement when making a decision, concerns regarding religious beliefs, pain management, and dependency (Karel et al., 2010).

After finishing the CAI-Health first version, a qualitative study with focus groups was conducted. Despite the lack of explicit assessment procedures in Portugal, it was likely that professionals had previously conducted or accompanied capacity assessment processes, which made explicit the gap between clinical practices and available research regarding the same. Also, despite the four abilities model being widely accepted and used, as mentioned, it has been based on United Sated legal standards of competence to consent. Given that an instrument for the Portuguese population was being developed, it was considered relevant to ensure that these abilities were valid for the Portuguese context. Holding these concerns as a starting point, qualitative research with focus groups was designed. This was deemed the best approach since it would allow for the exploration of individuals’ practices and perceptions regarding capacity assessment. Furthermore, as the research team indented to ascertain if the previous framework of four abilities was valid for the Portuguese context, the focus groups methodology was particularly suitable, since it is useful to amplify current theoretical knowledge, and to identify the most important variables when studying complex subjects, as is the case of decision-making capacity (Powell and Single, 1996). This qualitative research aimed to understand participants’ perception of healthcare decision-making capacity, discuss relevant aspects regarding the same capacity, identify assessment practices, present and examine CAI-Health, in order to identify new items or dimensions of interest.

## Materials and methods

The research protocol was reviewed and approved by the Ethic Commission of the University of Beira Interior (process number CE-UBI-Pj-2020-072:ID2172). The research design ensured participants’ anonymity. Considering the research goals, it was expected that participants might share some personal and professional experiences that should be kept confidential. Therefore, participants were asked not to disclose information shared during focus groups.

### Participants

To accomplish this research main goals, nine focus groups were conducted (*N* = 38) in 2020. To identify participants of interest, a theoretical sampling approach was followed, which means that researchers have identified characteristics that were likely to impact response variability based on previous knowledge (Powell and Single, 1996). Therefore, professionals in the fields of health, law, justice, and elder care, as well as older adults, were identified as interest groups.

Healthcare professionals (HP) were considered of interest for frequently facing situations that require thorough capacity assessments. Targeted professionals were psychologists, physicians, and nurses. To be included, participants should have work experience with older adults, excluding professionals specialized in children and younger adults. A group of recently graduated healthcare professionals was also planned. It was believed that recently graduated students could increase response variability, since their experience is mostly observational, and they frequently have a more critical view of common clinical practices.

Law and justice professionals (LJP) were targeted because the justice system is responsible for capacity determinations. The inclusion criteria allowed the participation of lawyers, judges, jurists, notaries, and conservators.

On the other hand, nursing home professionals (NHP) were identified as an interest group for their privileged work experience with the targeted population. Inclusion criteria were to have worked in the nursing home for more than 3 months and to be part of the nursing home’s technical or clinical team.

At last, older adults were also pointed out as an interest group, as they belong to the correspondent age range of the target population and shared the same cultural and historical background. Inclusion criteria were to have at least 60 years and not have a diagnosis of neurocognitive disease. Considering that a high percentage of older adults in Portugal live in nursing homes (Eurostat, 2019), it was considered relevant to have separated focus groups with institutionalized (IOA) and dwelling older adults (DOA).

Participants recruitment for HP and LJP focus groups were conducted through the identification and invitation of local and nationally recognized professionals in their work field. Regarding the NHP focus group, professionals from different geographical areas who had collaborated in previous research projects were invited to participate. Three participants of this group identified and established contact with residents from their nursing homes to participate in this research. Participants recruitment for DOA was conducted with support from a local network of the European Anti-Poverty Network. With the exclusion of older adults focus groups, all participants were invited *via* email. The research goals were fully described upon the invitation, as well as the voluntary character of the participation, and the possibility to cease the participation at any moment. Participants’ sociodemographic features from each group can be consulted in Table 1.

**Table 1:** Participants’ sociodemographic data.

	Healthcare professionals (HP)	Law/justice professionals (LJP)	Nursing home workers (NHP)	Institutionalized older adults (IOA)	Dwelling older adults (DOA)
*N*	11	4	4	15	4
Age	41.3 (*SD* = 16.5)	53.5 (*SD* = 10.4)	36.5 (*SD* = 3.1)	83.7 (*SD* = 10.1)	67.3 (*SD* = 3.3)
**Gender**					
Male	4 (36.3%)	1 (25%)		4 (26.6%)	1 (25%)
Female	7 (63.6%)	3 (75%)	4 (100%)	11 (73.3%)	3 (75%)
**Education**					
1–9 school years				12 (80.1%)	1 (25%)
High school				1 (6.7%)	
Licentiate	1 (9.1%)	3 (75%)	1 (25%)	2 (13.3%)	2 (50%)
Master	5 (45.5%)	1 (25%)	3 (75%)		1 (25%)
Ph.D.	5 (45.5%)				
**Profession**					
Physician	8 (72.7%)				
Neurology	1 (9.1%)				
Psychiatry	1 (9.1%)				
General Practice	1 (9.1%)				
Internal Medicine	1 (9.1%)				
Intensive Medicine	1 (9.1%)				
Oncology	1 (9.1%)				
Resident	2 (18.2%)				
Nurse					
Magister		1 (25%)			
Jurist		2 (50%)			
Conservator	2 (18.2%)	1 (25%)			
Psychologist			1 (25%)		
Technical director			3 (75%)		
Retired				15 (100%)	3 (75%)
Unemployed					1 (25%)

### Data collection

The focus groups were conducted online, through Zoom Platform. The reason to resort to online meeting was due to the National Government’s social distancing recommendations to prevent COVID-19 dissemination.

Two researchers were selected as mediators of the focus groups. Regarding the sessions management, focus groups with healthcare, nursing homes, and law and justice professionals followed the same structure: (1) Presentation of the session goals’, informed consent collection, and participants’ presentation; (2) Group discussion concerning healthcare decision-making capacity, based on semi-structured interview scripts; (3) CAI-Health presentation and analysis; (4) Suggestion of new items or dimensions; (5) Session closure. Focus groups with older adults both residents in institutions and the community included the same steps, with the exclusion of steps three and four, due to technical difficulties.

Semi-structured interview scripts were developed for each focus group. Interview scripts were previously presented and discussed with a group of experts. Despite having specific goals for each group, all scripts included core questions related to healthcare decision-making capacity definition and implied abilities, such as: *How do you define healthcare decision-making capacity? What aspects are implied in decision-making regarding healthcare?* Focus groups with healthcare and nursing home professionals included questions regarding capacity assessment, like: *In your professional context, have you ever conducted or followed an assessment of decision-making capacity in healthcare? What tools were used/would you use?* Law and justice professionals were asked questions about national legislation regarding decision-making, including: *Are there any legal standards that specify which characteristics are necessary for a person to be considered competent in healthcare decision-making?* Focus groups with older adults (institutionalizes and community residents) followed the same interview script, which focused mainly on participants’ personal experiences: *What aspects do you have in mind when you have to decide on your health? What aspects influence your capacity to make decisions regarding healthcare?*

Healthcare professionals were divided into three focus groups, due to schedule incompatibilities. One of them, which included three medical specialists, was asked to evaluate the clinical vignettes included in CAI-Health. Results regarding clinical vignettes will be presented elsewhere. Institutionalized older adults were also divided into three focus groups, due to the high number of participants. Therefore, nine focus groups were conducted: one group with the law and justice professionals, nursing home professionals and dwelling older adults, and three groups with healthcare professionals and institutionalized older adults.

After the session, participants were sent an online questionnaire to collect sociodemographic data. Nursing home collaborators sent sociodemographic data from older adults who participated in the focus group since they did not have the means to fill in online questionnaires. All older adults gave consent for formal caregivers to share their data. Focus groups lasted between 1 and 2 h and were digitally recorded. Group discussions were later fully transcribed.

### Data analysis

Data were analyzed through directed content analysis. This approach to qualitative analysis differs from the conventional analysis since the initial code definition is guided by theory. The directed content approach’s main advantage is that it allows confirmation and expansion of previous theoretical frameworks (Hsieh and Shannon, 2005). The authors had previously studied and worked with Grisso and Appelbaum’s (1998a) theory of abilities related to decision-making, which prevented them from looking at focus group data without preconceived notions regarding abilities implied in decision-making. Therefore, it was considered that the most straightforward approach to conducting the content analysis was to follow a directed methodology. However, the authors expected to identify data that did not match the initial coding scheme, since the identification of abilities related to decision-making capacity was only one of the research goals.

Content analysis began with the definition of initial coding categories, based on Grisso and Appelbaum’s (1998a) theory of abilities related to healthcare decision-making capacity. This process included the development of operational definitions of the four abilities identified in Grisso and Appelbaum’s (1998a) theory. The decision to conduct a direct content analysis, as well as the definition of initial coding categories were made by the research team. Regarding the following steps, it was decided that they would be conducted by two authors independently, following an analyst triangulation approach, to increase research credibility and confirmability (Shenton, 2004; Adler, 2022).

All transcripts were carefully reviewed, and all relevant text sections regarding decision-making capacity were highlighted. Next, highlighted sections were coded with pre-determined categories. Afterward, text sections that did not match pre-determined categories were given new ones. The option for highlighting all meaningful text sections and starting coding afterward was made to ensure that all content related to research goals was captured, increasing results trustworthiness (Hsieh and Shannon, 2005). When the coding process was finished, data associated with each category were checked, and subcategories were established when appropriate. Results of coding from the two authors were compared, and circumstances of non-agreement were presented and discussed with a third research team member. This content analysis process was conducted using the software MAXQDA2020 and involved multiple cycles of text and coding review until all researchers agreed on code attribution.

**Figure 1:**
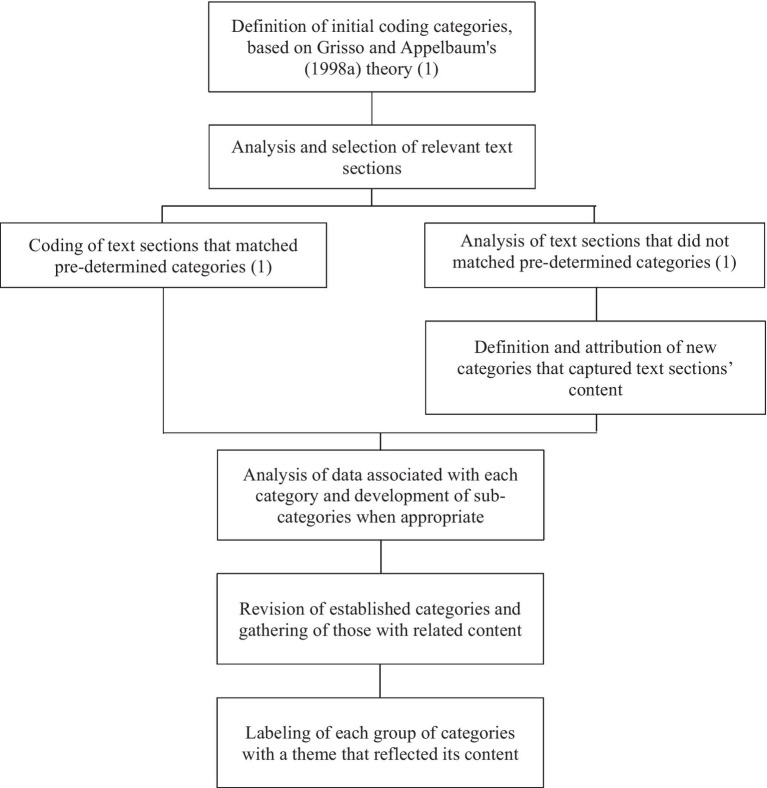
Diagram of data analysis process.

Finally, given the high number of categories and to facilitate the data interpretation, a framework of themes was developed by the research team. Within the content analysis, the attribution of themes allows for organizing and interpreting data content (Lindgren et al., 2020). This process was also conducted by two independent research team members. All categories and their data were examined. Categories with related content were grouped, and each was labeled with a theme that captured its essence. Outputs from both authors were compared, and non-agreement cases were reviewed with a third author. Results were discussed with all research members. The data analysis process is represented in Figure 1.

### Trustworthiness

The trustworthiness of data was measured according to Lincoln and Guba’s criteria of credibility, transferability, dependability and confirmability, as presented by (Shenton, 2004).

The development of semi-structured scripts, expert consultation, and the inclusion of participants with various backgrounds to increase response variability aimed to improve research credibility. Triangulation in data analysis, descriptions of the interview scripts and participants’ inclusion criteria detailed in this paper have the purpose to ensure confirmability as far as possible. Furthermore, the detailed descriptions of data collection and analysis also intend to address dependability, allowing the study to be repeated. In the same way, participants’ sociodemographic data, and theoretical and contextual framework were presented to enable other researchers to evaluate the suitability of comparisons that is, transferability.

## Results

From coded data interpretation, multiple themes related to healthcare decision making-capacity emerged. The number and scope of themes reflect the complexity of this concept and includes: (1) Decision-making capacity features; (2) Abilities implied in decision-making; (3) Factors that influence decision-making; (4) Obstacles to decision-making; (5) Legal aspects; (6) Assessment domains. These themes allow to identify professionals’ and older adults’ perceptions of healthcare decision-making capacity, as well as assessment domains and legal aspects related to this capacity in Portugal. Table 2 summarizes the results of the content analysis. A more detailed description of each theme can be found below.

**Table 2:** Content analysis results.

Theme	Category	Subcategory	Example/text excerpts
Decision-making capacity features	Self-determined		“it is a personal decision that the person has the right to make (…)” (LJP, 2)
	Specific		“the same person might be able to give consent to a determined act, but not have the capacity to consent to another one” (LJP, 2).
	Fluctuating		“there can be improvements and setbacks in our capacity” (LJP, 1)
Abilities implied in decision-making	Reasoning	Logical reasoning	“(…) the ability to think abstractly about several hypotheses in order to find a solution” (NHP, 4)
		Consequence’s foresight	“(…) perception of what the potential consequences of that decision are” (HP, 4)
		Alternative weighting	“to think what could be better in this balance of benefit against sacrifice” (HP, 11)
	Understanding		“capacity to understand what they are being told and what is being proposed to them” (LJP, 2)
	Appreciation	Acknowledgment of the health situation	“If the person knows what is happening, if he has a notion of the reality in which he is inserted, of the reality of the disease he is going through.” (HP, 4)
		Consequences of one’s decisions	“The patient has to know that he can decide what he wants for his health” (HP, 5)
		Professionals’ intentions	“I know they want to heal me, to help me feel better” (IOA, 1)
	Communicating a choice		“ability to verbally communicate these decisions” (LJP,2)
	Cognition		“being cognitively able to make a choice” (HP, 3)
Factors influencing decision-making	Family involvement		“whenever I have health issues, I have a close relative who is always consulted” (DOA, 4)
	Healthcare professional’s involvement		“I always want to know the doctor’s opinion, because we did not study to be a doctor” (IOA, 2)
	Emotional state		“if they are in a period of greater sadness, and depression, it is enough to condition their decision-making capacity” (NHP, 2)
	Literacy		“the person’s literacy in the area for which they are making the decision” (HP, 2)
	Financial overwhelm		“The financial part, the economic cost (…) in my experience, that is a very relevant aspect” (NHP, 1)
	Religious beliefs		“I never shared a tear when I discovered I had that health problem because I have a lot of faith in God” (DOA, 2)
	Dependency		“either you became disabled for the rest of your life and cannot work, or you become dependent, and you’ll go to a nursing home, or become dependent of your children, husband, or of someone who takes care of you” (DOA, 2)
	Personal history		“the patients’ beliefs, myths, fears and previous experiences” (HP, 7)
Obstacles to decision-making	Limitations to self-determination		“sometimes end up accepting things they did not want to accept because they are constrained in their freedom of decision” (LJP, 3)
	Stigma	Stigma related to disease	“you cannot just assume that a person who has Alzheimer’s disease or other dementia cannot make decisions regarding their health (…) What happens, in reality, is exactly that” (HP, 7)
		Stigma related to age	“for example with my aunt, who is 92 years old, professionals always assume that she does not have capacity, without performing any assessment” (LJP, 1)
	Communication		“some words that a lot of doctors use are too sophisticated for people to understand its meaning” (DOA, 2)
	Information access	Duty to inform	“the patient should have all the available information about the proposed procedure” (HP, 8)
		Right to information and non-information	“professionals should know precisely if the patient wants to hear about their diagnosis or not” (LJP, 2)
Legal aspects	Escort		“the person can be accompanied in their decisions and that some decisions can even be taken by the person accompanying them (…)” (LJP, 3)
	Informal Caregiver		“the Statute of the Informal Caregiver (…) give them the power to access the health information of the person they are taking care of” (LJP, 1)
	Informed Consent		“it is me who decides, and I do it through informed consent” (LJP, 1)
	Advance Directives	Health prosecutor	“(…) the person who must make the decision is the patient. If they cannot, it’s the health care proxy, if they have named one” (LJP, 1)
		Living will	“if due to transitory or definitive causes I am not in a position to give informed consent, the health professional must verify if I have made a living will and fulfill it” (LJP, 1)
Assessment domains	Cognitive assessment		“the Mini Mental State Examination, the Clock Drawing Test, and Montreal Cognitive Assessment” (HP, 9)
	Functionality assessment		“the Lawton Index and sometimes the Katz Index” (HP, 4)
	Emotional state assessment		“the Geriatric Depression Scale” (HP, 4)
	Life context		“have someone else to turn to and ask for additional information” (HP, 1)

### Theme 1: Decision-making capacity features

The first theme emerging from content analysis regarded participants’ perception of decision-making capacity in healthcare. Three categories are included in this theme: self-determined; specific; and fluctuating. Initially, self-determined was coded as “personal decision,” whereas fluctuating was firstly labeled as “progress and setbacks.” Decision-making is seen as self-determined since it is concerned as a personal decision that the person is entitled to. It is also specific to each capacity domain, meaning that “the same person might be able to give consent to a determined act, but not have the capacity to consent to another one” (LJP, 2). At last, participants considered decision-making capacity to be fluctuating. That means that capacity is not considered to be a static trait, but rather an aspect that can vary over time, and which does not always follow a linear process of loss. In this sense, participants mention that “there can be improvements and setbacks in our capacity” (LJP, 1). One participant gave an example of this fluctuating feature “I’ve had the chance to watch two different appointments of the same patient (…) in one of them the patient barely spoke, in that day he wasn’t capable of deciding, and on the other appointment he was talking, asking questions and was capable of making decisions” (HP, 7).

### Theme 2: Abilities implied in decision-making

This theme contains the categories derived from Grisso and Appelbaum’s (1998a) theory. However, one of the abilities identified, that is, one of the categories that emerged from the content analysis did not match the theoretical framework. Five main categories were identified: reasoning; understanding; appreciation; communicating a choice; cognition. Reasoning included the subcategories logical reasoning (referring to problem-solving skills), consequences foresight (for each alternative at hand), and alternative weighting (comparing both risks and benefits of each option). Understanding referred to the “capacity to understand what they are being told and what is being proposed to them” (HP, 2), therefore including understanding information related to the health status, as well as proposed treatments.

On what concerned appreciation, it involved acknowledging its own health situation, appreciating the consequences of one’s own decisions (recognizing its power to decide), as well as assessing health professionals’ intentions [“we need to trust our doctors” (IOA, 3), “I know they want to heal me, to help me feel better” (IOA, 1)]. Communicating a choice was clearly stated as the “ability to verbally communicate these decisions” (LJP, 2). Finally, cognition, which did not match initial coding categories, was stated as “being cognitively able to make a choice” (HP, 3), with HP identifying cognitive functions implied in decision-making, such as “executive functioning” (HP, 1).

### Theme 3: Factors influencing decision-making

Participants identified multiple factors that influence decision-making. These relate to internal or external aspects which may impact how patients make decisions, or which decisions they tend to make. This theme includes eight categories: Family involvement; Healthcare professional’s involvement; Emotional state; Literacy; Financial overwhelm; Religious beliefs; Dependency; and Personal history.

Family involvement relates to the patient’s wish to consult their relatives when a healthcare decision is required. This need was reported by both professionals from different areas [“the person prefers to have someone to help them decide” (HP, 4)], and by older adults “whenever I have health issues, I have a close relative who is always consulted” (DOA, 1). On the other hand, healthcare professionals’ involvement refers to the patient’s desire for advice from the healthcare professional. As in the previous category, this was expressed by both professionals from different areas, as well as older adults, as it can be seen in the following citations: “people frequently put the decision on the professional’s side” (NHP, 3); “Frequently the patient turns to us and says, ‘you know best, you are the one that should decide’” (HP, 9); “I always want to know the doctor’s opinion, because we did not study to be a doctor” (IOA, 2).

HP and NHP mentioned the impact of emotional state on decision-making, particularly regarding the negative impact of depressive states on decision-making capacity. In this respect, they mentioned that “if they are in a period of greater sadness, and depression, it is enough to condition their decision-making capacity” (NHP, 2).

Knowledge regarding health issues, “the person’s literacy in the area for which they are making the decision” (HP, 2), also emerged in content analysis. In addition to HP, older adults also mention literacy has been of great importance, as reflected here: “to have knowledge of things is very important in what concerns health” (DOA, 2).

The financial burden was mentioned by NHP after CAI-Health presentation and analysis. When asked if more items or dimensions should be added, participants specifically referred to this category: “The financial part, the economic cost (…) in my experience, that is a very relevant aspect” (NHP, 1).

Religious beliefs, initially coded as “faith” were outlined by both nursing home professionals and older adults. In the focus group with older adults, a participant described how their religious beliefs were important for them to accept the proposed treatment and believe they could recover: “I never shared a tear when I discovered I had that health problem because I have a lot of faith in God” (DOA, 2).

Dependency emerged in the older adults’ focus group analysis. Participants shared their views on how health issues must be timely addressed by each person, since in the opposite case “either you became disabled for the rest of your life and cannot work, or you become dependent, and you’ll go to a nursing home, or become dependent of your children, husband, or of someone who takes care of you” (DOA, 2). The last category, personal history, emerged within all focus groups with professionals from different areas. Participants believe that decision-making is also influenced by “the patients’ beliefs, myths, fears and previous experiences” (HP, 7).

### Theme 4: Obstacles to decision-making

Identifying obstacles to decision-making was not a goal of this research. Nonetheless, during data analysis, several categories emerged in relation to this theme across the different focus groups, except for IOA. The categories included in this theme are limitations to self-determination, stigma, communication, and information access.

Limitations to self-determination emerged in the analysis of NHP and LJP focus group transcriptions. It describes situations where the patient’s right to decide about their healthcare is compromised by external factors. These can relate to “being influenced in some way” (LJP, 4), or “the time that is given to the person” (NHP, 3), resulting in situations where patients “sometimes end up accepting things they did not want to accept because they are constrained in their freedom of decision” (LJP, 3).

Stigma emerged within the HP and LJP focus groups and relates to negative preconceived beliefs that healthcare professionals hold toward some patients. This category includes stigma related to disease “you cannot just assume that a person who has Alzheimer’s disease or other dementia cannot make decisions regarding their health. That does not make sense to me. What happens, in reality, is exactly that” (HP, 7), as well as the stigma associated with age “for example with my aunt, who is 92 years old, professionals always assume that she does not have capacity, without performing any assessment” (LJP, 1).

The two last categories are communication and information access. The first was observed in all research groups, and it relates to doctor-patient communication. Initially, this category had been coded as “technical language” and “speech complexity,” until it was labeled *communication*. Participants described how healthcare providers’ communication is frequently inadequate, considering the patients’ scholarly level or language skills. A participant from the DOA group indicated that “some words that a lot of doctors use are too sophisticated for people to understand its meaning” (DOA, 2). In this context, it was mentioned in the HP group that “we physicians often use terms which are absolutely undecipherable for patients” (HP, 10). A participant from the same group pointed out as a possible aggravating factor that “I believe that none of us was taught on communication skills during university education” (HP, 11).

The last category, information access, includes the duty to inform and the right to information and non-information. On one hand, all focus groups reflected the idea that “the patient should have all the available information about the proposed procedure” (HP, 8). On the other hand, LJP also pointed out that “professionals should know precisely if the patient wants to hear about their diagnosis or not” (LJP, 2), since the law states that patients can refuse to be informed about their diagnosis.

### Theme 5: Legal aspects

The categories that emerged within this theme were: Escort; Informal Caregiver; Informed Consent; and Advance Directives. Identifying and discussing legal questions and procedures associated with healthcare decision-making capacity was a specific goal of the focus group with LJP. Therefore, only one subcategory (living will) emerged from a different focus group.

The first two categories, *escort* and *informal caregiver*, concern two different figures of the Portuguese Justice System. An escort may be requested to Court if an adult finds themselves unable to fully exercise their personal rights or fulfill their duties. In such cases, the escort allows that “the person can be accompanied in their decisions and that some decisions can even be taken by the person accompanying them, or by the people accompanying them, there can be several, depending on the decision areas that require assistance” (LJP, 3). Regarding the escort’s power to make healthcare decisions on behalf of the patient, one participant reported “what the Status of the Accompanied Adult (*Estatuto do Maior Acompanhado*) states is that, unless otherwise specified, personal rights continue to be exercised by the individual (…) our health is a personal right” (LJP, 2). In contrast, concerning the informal caregiver, participants described that “the Statute of the Informal Caregiver does not give the caregiver authorization to make health decisions, but it does give them the power to access the health information of the person they are taking care of” (LJP, 1).

Informed consent referred to the legal instrument through which people can decide about their health “it is me who decides, and I do it through informed consent” (LJP, 1). Finally, advance directives included health prosecutor and living will. The health prosecutor is the legal figure who can substitute the patient in the decision-making process “it’s very clear for me, from a legal point of view, that the person who must make the decision is the patient. If they cannot, it’s the health care proxy, if they have named one” (LJP, 1). In its turn, the living will be of use “if due to transitory or definitive causes I am not in a position to give informed consent, the health professional must verify if I have made a living will and fulfill it” (LJP, 1). This was the only subcategory of this theme that also emerged in the analysis of NHP transcription.

### Theme 6: Assessment domains

Identifying capacity assessment domains was defined as one of the main goals of HP focus groups. Therefore, the categories in this theme emerged from the transcript analysis of the HP discussion. The following assessment domains were identified: cognitive assessment; functional assessment; emotional state assessment; and life context.

With respect to cognitive assessment, participants reported the use of cognitive screening tests, such as “the Mini Mental State Examination, the Clock Drawing Test, and Montreal Cognitive Assessment” (HP, 9). On what concerned functional assessment, regarding specific assessment tools, participants reported using “the Lawton Index and sometimes the Katz Index” (HP, 10). Targeting emotional state assessment, participants indicated “the Geriatric Depression Scale” (HP, 10). The last category, regarding life context, reflected the resource to informants “have someone else to turn to and ask for additional information” (HP, 1) and gathering information concerning living conditions.

## Discussion

This study uses a qualitative methodology, which is an innovation in the study of healthcare decision-making capacity. Research results seem to validate and extend Grisso and Appelbaum’s theory, indicating a new ability implied in decision-making, as well as a new conceptualization of *appreciation*, which might be specific for older adults. In addition, this study allowed to identify specific obstacles to older adults’ capacity to make healthcare decisions. This finding has direct implications for clinical practice, as it allows the conception of preventive measures that may promote older adults’ capacity.

The themes that emerged reveal that decision-making is a complex concept, involving multiple abilities, psychological factors, as well as external influences (Ratcliff, 2020; Gaubert and Chainay, 2021). Furthermore, healthcare capacity assessment is based upon some established practices and has as background the country’s legal statutes (Ratcliff, 2020). Broadly, these results seem to confirm previous theoretical formulations regarding capacity. Concerning healthcare, capacity refers to the ability to decide what happens to one’s body, which is a personal right (Palmer and Harmell, 2016). Therefore, “it is a personal decision that the person has the right to make (…)” (LJP, 2). Consent capacity is also considered to be specific to the decision that the person is facing at the moment (Scott, 2008), as stated by participants. Although capacity assessments require a dichotomous determination, this ability frequently varies in a continuous way, with patients sometimes being able to make medical decisions regarding routine procedures, but not decisions involving high-risk treatments (ABA and APA, 2008). In this respect, the capacity course of evolution may also be affected by cognitive fluctuations, which can result in capacity changes (Trachsel et al., 2015; Triebel et al., 2018), as stated by participants.

Content analysis results support Grisso and Appelbaum’s (1998a) four abilities model, which discriminates four functional abilities implied in healthcare decision-making: communicating a choice; understanding; appreciation; and reasoning. Communicating a choice and understanding, as identified in content analysis, are a clear reflection of theoretical definitions. Appreciation seems to be related to the person’s beliefs regarding their health state, their role in decision-making, and beliefs regarding the intentions of those providing care. Beliefs regarding health state seem to meet Grisso and Appelbaum’s (1998a) description of appreciation, according to which appreciation involves the ability to apply information to one’s own situation. Beliefs concerning professionals’ intentions have also been contemplated in previous assessment instruments as part of appreciation (e.g., Etchells, 1996; Moye et al., 2007). However, appreciating the consequences of one’s own decisions, which reflects the recognition that one has the power and autonomy to make decisions about their health is, to our knowledge, a new conceptualization of this concept. To our understanding, this subcategory emerged due to the research focus on older adults. This particular section of the population grew up with a paternalistic healthcare culture, with older adults recognizing the physician as an unquestionable authority (Wetzels et al., 2004). Therefore, some people might believe that they do not have the right to choose between treatment options, nor to reject a treatment proposal. Due to this result, a new item was added to the capacity assessment interview. Furthermore, it is considered that this operationalization should be addressed when assessing capacity in older adults. In what concerns reasoning, the identified subcategories meet previous descriptions, that define reasoning as involving logical information processing, comparing the risks and benefits of each alternative, as well as foreseeing consequences on daily living (Grisso and Appelbaum, 1998a). It should be noticed that these results reinforce foresight as a reasoning ability, which contradicts some conceptualizations that address it as part of appreciation (Amaral et al., 2021).

In addition to the four abilities in Grisso and Appelbaum’s (1998a) model, results also point to cognition as an ability implied in decision-making. Previous research has tried to identify cognitive predictors of healthcare decision-making (e.g., Okonkwo et al., 2008; Stormoen et al., 2014; Darby and Dickerson, 2017). Three core cognitive tasks have been highlighted: information comprehension and encoding; processing information and reaching a decision; and communicating a decision (Marson and Hebert, 2011). These rely on short-term memory, receptive language, information processing, executive functioning, and expressive language (Marson and Hebert, 2011; Palmer and Harmell, 2016). The emergence of this category is interpreted as representing the need to perform a cognitive assessment when undergoing capacity evaluations.

Regarding factors influencing decision-making, all categories were already included in CAI-Health, with exception of financial overwhelm, emotional state, literacy, and personal history. Family and healthcare professionals’ involvement, religious beliefs, and dependency have been identified in previous studies about decision-making (Karel et al., 2007; Ratcliff, 2020), and were contemplated in CAI-Health as part of the questionnaire of healthcare values and preferences. The financial burden was added to the questionnaire after finishing this study. Despite not being included, emotional state is considered by the research team to be a relevant variable when assessing capacity. Therefore, similarly to cognition, emotional state, particularly the presence of depressive symptoms, should be addressed with a specific tool when assessing capacity, since it can negatively affect it (Hindmarch et al., 2013). Although literacy can impact the way patients understand information, low literacy cannot be a cause for incapacity, since that would lead us back to a paternalistic model of care, where professionals were assumed to know best what was better for patients (Fernández-Ballesteros et al., 2019). With respect to personal history, life experiences can significantly affect the way people decide regarding their health (Ratcliff, 2020). For example, research indicates that caregivers of patients with degenerative diseases are less likely to accept live-prolonging measures (Moye et al., 2006). It is our believe that life events and/or experiences that impact people’s beliefs regarding health and quality of life should be collected during the clinical interview, as part of capacity assessment.

The emergence of obstacles to decision-making had not been established as a research goal, but it is coherent with previous literature which identifies professionals’ bias as a possible barrier to decision-making (Fenge and Lee, 2020). Generally, this theme identified two areas in which professionals should have more training, specifically, decision-making capacity and communication skills. Further considerations regarding these results will be presented in the research implications. In the legal frame, the recent Law of Accompanied Adult (Decree-law 49/2018, 2018) evidences the growing concern with respecting peoples’ right to self-determination and protection. The fact that the areas in need of support can be determined, as well as the provision of ending the accompanying if the causes that determined it modify, meets the theoretical conceptualizations of decision making as specific and changeable over time (ABA and APA, 2008; Scott, 2008). However, in contrast with other countries (e.g., United Kingdom and United States), it stands out the absence of legal guidelines regarding abilities required for a person to be considered competent, and the opportunity to develop healthcare decision-making capacity as a new juridic categoric within the Portuguese justice system (Pereira, 2016).

On what concerns the assessment domains contemplated by participants, it is of interest to note that cognition and emotional state, that emerged in other themes, are also contemplated here. To our understanding, this corroborates our view that these aspects should not be part of a tool to evaluate capacity but included in a broad protocol that should be undertaken during a capacity assessment. One limitation that stood out was the lack of a specific instrument to evaluate capacity. Although participants had a variety of tools to evaluate variables related to capacity, they mentioned the need for a specific instrument to assess decision-making. In this respect, participants from professional groups highlighted the pertinence of developing CAI-Health, considering it adequate to evaluate capacity and manifest interest to use it in their practice.

When examining these research findings, some limitations must be taken into consideration. First, concerning the sample size, focus groups should ideally be composed of six to a maximum of 15 participants (Powell and Single, 1996; Eaton, 2017). In this research, focus groups with nursing home professionals, law and justice professionals, and dwelling older adults only included four participants, which can be a limitation to research dependability., It is also relevant to point out the method by which focus groups were conducted, since some participants could have felt more comfortable in person. Furthermore, some issues with sound and network signal in nursing homes created some challenges in sessions with institutionalized older adults. However, it is considered that this approach is also one of the strengths of this research since online meetings made it possible to assemble professionals from different geographical areas.

Despite mentioned limitations, it is considered that research results have implications for clinical and assessment practices. From a clinical perspective, considering that cognitive fluctuations can impact capacity, information’s regarding healthcare issues and treatment options should be given at the time when the older adult is most likely to be alert and oriented. When appropriate, healthcare providers should also ensure that the person recognizes that they can choose between adequate treatment options, as well as refuse treatment. Regarding assessment procedures, research results indicate that capacity assessments should rely on a comprehensive protocol. These would include assessment tools of cognitive functioning, depressive symptoms, functionality, and decision-making abilities. At last, preventive strategies to promote older adults’ autonomy can also be drawn from research findings. These include actions to increase health literacy among older adults and health providers’ knowledge of decision-making capacity and communication skills. Since literacy influences people’s ability to understand information, measures to increase older adults’ literacy on health could promote their capacity to make decisions. On what concerns healthcare providers, first, health professionals should have more knowledge regarding decision-making capacity. This would allow decreasing frequent misconceptions regarding age, neurocognitive disorders, and their relationship with capacity (Donnelly et al., 2019), and consequently diminish stigma. Similarly, training on decision-making would promote education about patients’ rights to receive information, as well as increase professionals’ competencies to involve patients in decision-making processes (Légaré and Witteman, 2013). Second, health professionals, particularly physicians, would also benefit from more training in communication skills, as expressed by both older people, as well as health practitioners. Communication skills are essential to clinical practice (Hain and Saad, 2016), and should therefore be properly developed and trained by professionals. Furthermore, poor communication skills can impact decision-making capacity, since it can affect patients’ understanding of information (King and Hoppe, 2013).

## Conclusion

Decision-making capacity outlines go further than assessment procedures, dwelling around ethics, cultural beliefs, healthcare guidelines and practices, legislation, and education. It is important to notice that these study results represent the perspectives and practices of a restricted number of Portuguese professionals and older adults. In this respect, it would be of interest to replicate this research with other populations, in order to compare and identify variations in assessment practices. One important contribution of this research is the identification of practical aspects that can be conducted to promote healthcare decision-making capacity. These relate to the areas in which health professionals need more training, which could be accomplished by including communication skills and capacity issues in university curricula. However, it appears vital to go further and improve society’s literacy regarding capacity, enhancing people’s knowledge about their rights and means to protect those with compromised capacity.

Regarding CAI-Health, the capacity assessment interview, based on the four abilities model (Grisso and Appelbaum, 1998a) meets the abilities identified by participants as implied in decision making. Furthermore, our results revealed a new conceptualization of appreciation, as one’s recognition of its role and power in the decision-making process. On what concerns the questionnaire of health care values and preferences, besides the item related to the financial burden (proposed by participants) focus group results corroborate the variables included in this tool. Therefore, it is considered that the research results validate CAI-Health proposed content and structure.

## Data availability statement

The datasets presented in this article are not publicly available (because participants were asked to consent to the processing of data only within the scope of the specific objectives of this investigation) but are available from the corresponding authors upon reasonable request. Requests to access the datasets should be directed to anapaula@fcsaude.ubi.pt.

## Ethics statement

The studies involving human participants were reviewed and approved by Ethical Committee of University of Beira Interior (reference CE-UBI-Pj-2020-072). Written informed consent for participation was not required for this study in accordance with the national legislation and the institutional requirements.

## Author contributions

AA and RA were responsible for facilitating the focus groups. AA transcripted group discussions and wrote the manuscript in consultation with MS, SF, MV, LS, and RA. AA, MS, SF, MV, LS, and RA conducted data analysis. All authors contributed to the article and approved the submitted version.

## Funding

AA is a PhD student at Faculty of Psychology and Educational Sciences, University of Coimbra, supported by Fundação para a Ciência e a Tecnologia (FCT), with an Individual PhD Grant (FCT, SFRH/BD/138897/2018), financed by national funds from the Ministério da Ciência, Tecnologia e Ensino Superior (MCTES) and the European Social Fund (ESF-EU) through Programa Operacional Regional do Centro (PORC-EU). University of Coimbra supported open access publication fees.

## Conflict of interest

The authors declare that the research was conducted in the absence of any commercial or financial relationships that could be construed as a potential conflict of interest.

## Publisher’s note

All claims expressed in this article are solely those of the authors and do not necessarily represent those of their affiliated organizations, or those of the publisher, the editors and the reviewers. Any product that may be evaluated in this article, or claim that may be made by its manufacturer, is not guaranteed or endorsed by the publisher.
